# Mass Gathering Events at an Event Hall in Osaka Are a Non-direct Risk of Admission to a Neighboring Emergency Hospital

**DOI:** 10.7759/cureus.65410

**Published:** 2024-07-26

**Authors:** Hiroshi Fukuoka, Hiroshi Matsuura, Hideo Yanagi, Jun Oda

**Affiliations:** 1 Emergency Department, Tane General Hospital, Osaka, JPN; 2 Emergency and Critical Care Center, Osaka Prefectural Nakakawachi Medical Center of Acute Medicine, Osaka, JPN; 3 Traumatology and Acute Critical Medicine Department, Osaka University Graduate School of Medicine, Osaka, JPN

**Keywords:** risk factor, planned event, penumbra, mass gathering, admission

## Abstract

Background: The influence of planned mass gathering events on surrounding residents is not understood sufficiently.

Objectives: The purpose of this study was to investigate how events at an event hall affect a neighboring emergency hospital throughout the year.

Methods: This was a single-center, retrospective, observational study conducted on all patients who presented to the emergency department from January 1 to December 31, 2019. The event hall is located 200 meters from the hospital, and various events such as music concerts and professional baseball games are held at the hall throughout the year. We collected patient information from the electronic medical records. The factors associated with hospitalization were assessed using a multivariable logistic regression analysis.

Results: This study comprised 18,907 patients who visited our emergency department. The number of patients on event days was 9,981 and that on no-event days was 8,922. The mean (SD) number of patients visiting on event days was 56.4 (14.9), and that on no-event days was 47.5 (14.1) (p<0.05). The multivariable logistics regression analysis showed age (adjusted odds ratio (AOR): 1.03; 95% confidence interval (CI): 1.03-1.04), male gender (AOR: 1.21; 95% CI: 1.13-1.31), transportation by emergency medical services (AOR: 2.56; 95% CI: 2.37-2.75), rain days (AOR: 1.14; 95% CI: 1.04-1.23), and event day (AOR: 1.11; 95% CI: 1.02-1.20) to be independent risk factors of hospitalization.

Conclusions: In this study, we found that event day was one of the independent risk factors of admission to the hospital from the emergency department.

## Introduction

The World Health Organization defines mass gathering as the concentration of people at a specific location and period for a specific purpose, which has the potential to strain the planning and response resources of the host community or region where it is being held [1]. Although some studies reported patient characteristics from mass gathering events and their impact on hospitals and emergency services [2-4], only a few studies have reported on the influence that such events have on surrounding residents who require medical care but were not involved in mass gathering events. These include such effects such as an increase in mortality rates of patients due to acute myocardial infarction or cardiac arrest that occurred in the vicinity during major marathons [5], changes in emergency transport times during the G20 summit in Osaka [6], and an increase in emergency calls in an event area in Finland [7]. During event periods, the population of participants and event staff increases rapidly but temporarily. Therefore, the number of patients attending a neighboring emergency hospital may also increase. An event held in an event hall is a planned mass gathering, and the results of this study may be able to predict the influence of mass gathering events on neighboring hospitals. Therefore, the objective of this study was to investigate how mass gathering events affect a neighboring emergency hospital throughout the year.

## Materials and methods

Study design and setting

**Figure 1:**
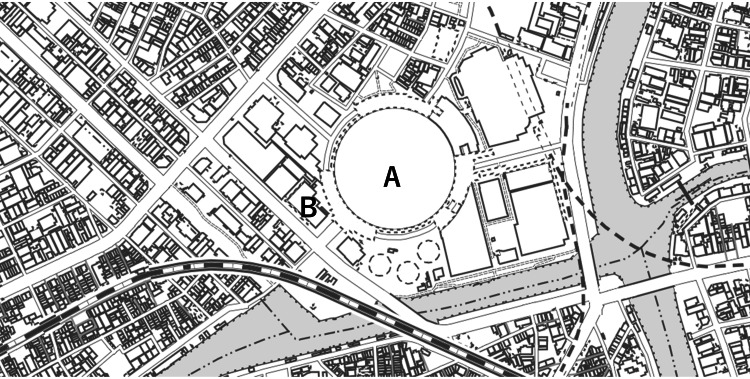
Map of the event hall and the neighboring hospital. The hospital is 200 meters on foot from the event hall. A: The event hall. B: Tane General Hospital. This map is from the Geographical Survey Institute in Japan provided free of charge (https://www.gsi.go.jp/kiban/).

This was a single-center, retrospective, observational study conducted on all patients who presented to our emergency department (ED) from January 1 to December 31, 2019. The ED is in a private general hospital handling approximately 19,000 patient visits per year and accepts almost all patients from nearby events. This observational study followed the principles of the Declaration of Helsinki and was approved by the Institutional Review Board for Clinical Research of the hospital (approval no.: 2022-13). The event hall is located 200 meters from the hospital, and various events such as music concerts and professional baseball games are held throughout the year (Figure 1).

The indoor event hall has a ceiling height of 60 m, an arena area of 13,200 square meters, an approximate volume of 1,200,000 cubic meters, and a maximum capacity of 55,000 persons. Seats and air conditioning are provided. The event hall’s restaurants are open when the events are held, and alcohol is served depending on the events. Medical staff including doctors and nurses who triage the patients in the event hall are always in attendance. If the patients need to visit the hospital, medical staff arrange for transportation of the patients.

Data collection

We collected patient information from the electronic medical records, which included demographic characteristics, hospitalization, hours of consultation, day of arrival, transportation to the ED by emergency medical services (EMS), event day or no-event day, weather, and relation to the event. Holidays included Sundays, national holidays, and the periods from January 1 to 3, and from December 29 to 31. After-hour consultation was defined as that occurring from 0:00 to 9:00 and 17:00 to 23:59 on weekdays and from 0:00 to 9:00 and 12:00 to 23:59 on Saturdays. Sundays and holidays are the overtime after-hours consultation. Rain day was defined as a day with 0.5 mm or more of precipitation from 0:00 to 23:59, based on weather data observed by the Automated Meteorological Data Acquisition System in Osaka City obtained from the website of the Japan Weather Association. Event days and no-event days were determined based on the official website of the event hall. Closed events were treated as no-event days. Indication for admission was determined by the ER physician.

Main outcome

The primary outcome was the assessment of the risk of admission to the hospital from the ED on event and no-event days. The secondary outcomes were the number of patients seen in the ED on event days and no-event days and the characterization of the patients who came from the event hall.

Statistical analysis

The number of patients and patient age are expressed as mean (standard deviation (SD)). Other data are expressed as counts and percentages. The Student t-test and Fisher’s exact test were used to compare the patient characteristics on event days and no-event days. The factors associated with admission were evaluated by multivariate logistic regression analysis, and the adjusted odds ratio (AOR) and 95% confidential interval (CI) were calculated. In the logistic regression model, we included age, gender, after-hours consultation, holidays, use of EMS, event days, rain days, and traumatic injury as the variables. These variables were first considered as variables for analysis by comparing all data between event and non-event days. A p value <0.05 was considered to be statistically significant. Statistical analyses were conducted with JMP Pro (version 16.2 for Windows; SAS Institute Inc., Cary, NC). This manuscript was written based on the strengthening the reporting of observational studies in epidemiology (STROBE) statement to assess the reporting of cohort and cross-sectional studies [8].

## Results

Patient characteristics

**Figure 2:**
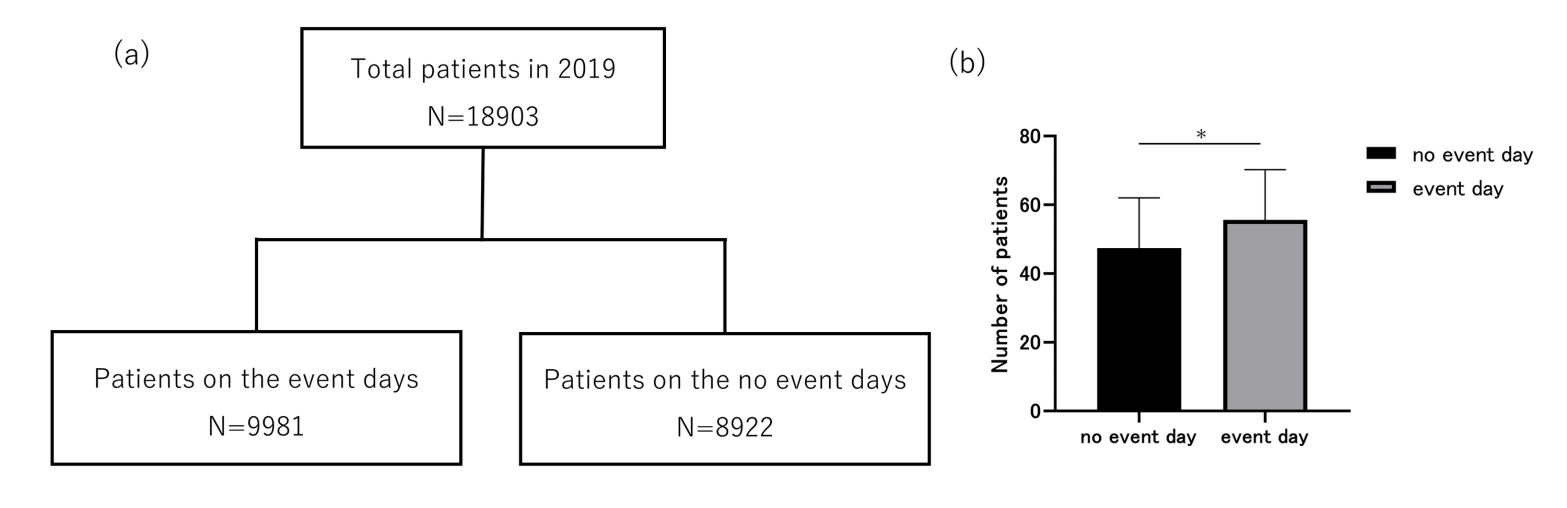
Patient flow diagram (a) and mean number of patients visiting the emergency department (b) on event days and no-event days. The whisker bars represent the standard deviation. *P<0.05

This study comprised 18,907 patients who visited our ED between January 1 and December 31, 2019. The number of patients visiting on event days was 9,981 (3,571 on weekdays and 6,410 on holidays), and that on no-event days was 8,922 (7,427 on weekdays and 1,495 on holidays) (Table 1, Figure 2).

**Table 1:** Characteristics of the ER patients in Tane Hospital in 2019.

	Total		Event Days		No-Event Days		
							P
Characteristics	(N = 18,903)		(N = 9981)		(N = 8922)		
Days, n (%)	365	(100%)	177	(48.5%)	188	(51.5%)	0.276
Holidays, n (%)	124	(34%)	103	(83.1%)	21	(16.9%)	<0.001
Wet-days, n (%)	107	(29%)	54	(50.5%)	53	(49.5%)	0.647
Patients, n (%)							
Holidays, n (%)	7905	(41.8%)	6410	(64.2%)	1495	(16.8%)	<0.001
Wet-days, n (%)	5473	(29.0%)	6482	(64.9%)	2440	(27.4%)	<0.001
Average of patients, mean (±SD)	51.8	(±14.4)	56.4	(±14.9%)	47.5	(±14.1)	<0.001
Age, average (±SD)	54.6	(±25.3)	56.5	(±25.5%)	55.1	(±25.0)	<0.001
Sex, n (%)							
Female, n (%)	9226	(48.8%)	4997	(50.1%)	4229	(47.4%)	<0.001
After-hours consultations, n (%)	14,320	(75.8%)	8330	(83.5%)	5992	(67.2%)	<0.001
From EMS, n (%)	8205	(43.4%)	4184	(41.9%)	4021	(45.1%)	<0.001
Traumatic injury, n (%)	5551	(30.4%)	3112	(31.2%)	2537	(28.4%)	<0.001
Hospitalization, n (%)	5332	(28.2%)	2679	(26.8%)	2653	(29.7%)	<0.001

The patients’ characteristics are summarized in Table 1. The number of rain days was 107 (54 event days and 53 no-event days). The number of patients visiting the ED per day was 51.8 (14.4), with 56.4 (14.9) visiting on event days and 47.5 (14.1) visiting on no-event days. The number of patients who visited for after-hours consultation was 14,320 (8,330 on event days and 5,990 on no-event days), and the number of hospitalizations was 5,332 (2,679 on event days and 2,653 on no-event days) (Table 1). On event days, the average number of patients, after-hours consultations, and traumatic injuries were larger than those on no-event days. The number of patients transported by EMS and who were hospitalized was larger on no-event days.

Factors associated with hospitalization

We assessed the factors associated with hospitalization using a multivariable logistic regression analysis in a model that included age, sex, after-hours consultation, transportation by EMS, holidays, rain days, events, and traumatic injury. Age (AOR: 1.03; 95% CI: 1.03-1.04), male gender (AOR: 1.21; 95% CI: 1.13-1.31), transportation by EMS (AOR: 2.56; 95% CI: 2.37-2.75), rain days (AOR: 1.14; 95% CI: 1.04-1.23), and event day (AOR: 1.11; 95% CI: 1.02-1.20) were the independent risk factors of hospitalization (Table 2).

**Table 2:** Factors associated with hospitalizations. AOR, adjusted odds ratio. CI, confidence interval. EMS, emergency medical service.

	AOR	95% CI	P
Age	1.03	(1.03-1.04)	<0.001
Sex (male)	1.21	(1.13-1.31)	<0.001
After hour consultation	0.61	(0.56-0.66)	<0.001
From EMS	2.56	(2.37-2.75)	<0.001
Holidays	0.80	(0.74-0.88)	<0.001
Wet-days	1.14	(1.04-1.23)	0.002
The days with events	1.11	(1.02-1.20)	0.013
Traumatic injury	0.27	(0.25-0.30)	<0.001

Patients from the event hall

During the study period, 143 patients from the event hall presented to the ED (49 patients visited on weekdays and 94 patients visited on holidays). Their age was 37 (19.9) years old. Among these patients, 57 were male and 86 were female. The number of patients visiting for after-hours consultation was 134 (93.7%), that using EMS was 57 (39.9%), and that of trauma patients was 56 (39.2%). Among them, 16 (11.2%) patients were hospitalized (Table 3).

**Table 3:** Characteristics of the ER patients from the event hall in 2019.

	Total	
Characteristics			(N = 143)	
Patients, n (%)		
Weekdays, n (%)	49	(34.3%)
Holidays, n (%)	94	(65.7%)
Age, average (±SD)	37	(±19.9)
Sex, n (%)		
Female, n (%)	86	(60.1%)
After-hours consultation, n (%)	134	(93.7%)
From EMS, n (%)	57	(39.9%)
Traumatic injury, n (%)	56	(39.2%)
Hospitalization, n (%)	16	(11.2%)

We found no significant difference in patient characteristics based on the type of event attended.

## Discussion

In this study, we found that the average number of patients visiting the ED on event days was larger than that on no-event days, and event days were one of the independent risk factors of admission to the hospital from the ED. In a previous study, holiday was an independent factor in the increasing number of patients visiting an ED [9]. The event hall in this study hosted mass gathering events on about half of the dates during the year, and many open events were held on holidays. Thus, the increase in the number of patients on event days may be caused in part by some days being holidays. We also showed that the independent risk factors of admission to the hospital of patients visiting the ED were age, male gender, transportation by EMS, rain days, and event days. Generally, the patients transported by EMS were more severely ill or injured than those walking into the ED, and similar results have already been reported [10]. Moreover, aging and male gender have been reported as risks of admission [11]. Rain days correlated positively with the use of EMS [12], and our results showed these days to be an independent risk factor of admission. An increase in patients transported by EMS may cause increased admissions through an ED.

The rate of admission of patients who visited the ED from the event hall was lower than the overall rate of admission, and the number of patients from the event hall was limited. Thus, it is unlikely that events at the event hall themselves were a direct risk factor for admission of patients visiting the ED, but mass gathering events may influence “penumbra” and increase the risk of hospitalization from the ED of a hospital near the event hall. The “penumbra” is defined by Lund et al. [13], as crowds (non-ticket holders, fans, and protesters) gathering outside the boundaries of the event and as local residents whose basic health services are affected by the disruption of services related to the event. However, penumbra is difficult to assess [13]. There are some previous studies on penumbra being influenced by mass gathering events. First, a large marathon event had an influence on the penumbra by delaying ambulance transport times and increasing the mortality rate of patients with acute myocardial infarction and cardiac arrest that occurred in the neighborhood [5]. Second, the EMS in Finland reported an increase in the need for EMS in the event region due to violence, traffic trauma, alcohol-related illnesses, and other accidents or injuries during the event period, whereas the need for EMS due to other diseases had not been increased [7]. Although it is difficult to clearly explain the cause of an increased risk of hospitalization on event days, a rapid short-term increase in the local population caused by a mass gathering event may have various effects on the penumbra, resulting in a multifactorial risk of admission to the hospital.

The risk factors for the increasing number of patients at mass gathering events are outdoor and unbounded venues, the absence of free water (i.e., that provided free of charge), the lack of climate control, the percent of occupied seating, an increasing heat index, and alcohol and drug use [3,14-16]. The event hall in this study is indoors and has climate control and seating. In addition, the use of drugs is prohibited in Japan, and alcohol use was limited during this study period (and in some cases, it is not sold at events). When comparing the characteristics of patients visiting the ED from the event hall with all patients visiting the ED, more young people, more women, and more after-hours consultations were detected from the event hall.

More patients from the event hall had mild traumatic injury as their main complaint, and a small number of these patients required hospitalization. Thus, patients from the event hall in this study were not a direct risk of increasing admission to the hospital. An increase in trauma around event venues in Finland [7] and the high incidence of patients with traumatic injury from the event hall in the present study were considered to be a similar result. In addition, although previous reports have indicated that the type of event and the temporary state of feeling of the participants can influence the incidence of injuries and illnesses [14,17-19], the influence of the type of event on hospital admissions showed no significant difference in the present study.

Limitations

We acknowledge several limitations of our study. First, the retrospective single-center design and short study duration may have influenced the precision of our findings. Second, the hospital did not accept all patients from around the event hall, and this may limit the hall’s influence. Third, as this is an observational study, there may be unknown confounding factors. Finally, this study assessed planned mass gatherings. Thus, other events such as spontaneous mass gatherings were not included nor discussed. Further research is needed to clarify and resolve these limitations.

## Conclusions

Planned mass gathering events held at the event hall were found to be an independent risk factor for the admission of patients surrounding the venue to the ED at the neighboring emergency hospital. Thus, planned mass gathering events may affect a neighboring emergency hospital throughout the year, and the present results may be useful in establishing an EMS for neighboring hospitals.
